# Advancements in mitochondrial-targeted nanotherapeutics: overcoming biological obstacles and optimizing drug delivery

**DOI:** 10.3389/fimmu.2024.1451989

**Published:** 2024-10-17

**Authors:** Yang Li, Xiao-meng Li, Li-si Wei, Jun-feng Ye

**Affiliations:** ^1^ General Surgery Center, First Hospital of Jilin University, Changchun, China; ^2^ Department of Rehabilitation, School of Nursing, Jilin University, Changchun, China

**Keywords:** mitochondrial targeting, nanotechnology, drug delivery systems, subcellular organelles, therapeutic nanocarriers, biocompatibility

## Abstract

In recent decades, nanotechnology has significantly advanced drug delivery systems, particularly in targeting subcellular organelles, thus opening new avenues for disease treatment. Mitochondria, critical for cellular energy and health, when dysfunctional, contribute to cancer, neurodegenerative diseases, and metabolic disorders. This has propelled the development of nanomedicines aimed at precise mitochondrial targeting to modulate their function, marking a research hotspot. This review delves into the recent advancements in mitochondrial-targeted nanotherapeutics, with a comprehensive focus on targeting strategies, nanocarrier designs, and their therapeutic applications. It emphasizes nanotechnology’s role in enhancing drug delivery by overcoming biological barriers and optimizing drug design for specific mitochondrial targeting. Strategies exploiting mitochondrial membrane potential differences and specific targeting ligands improve the delivery and mitochondrial accumulation of nanomedicines. The use of diverse nanocarriers, including liposomes, polymer nanoparticles, and inorganic nanoparticles, tailored for effective mitochondrial targeting, shows promise in anti-tumor and neurodegenerative treatments. The review addresses the challenges and future directions in mitochondrial targeting nanotherapy, highlighting the need for precision, reduced toxicity, and clinical validation. Mitochondrial targeting nanotherapy stands at the forefront of therapeutic strategies, offering innovative treatment perspectives. Ongoing innovation and research are crucial for developing more precise and effective treatment modalities.

## 1 Introduction

The field of nanomedicine has witnessed a significant paradigm shift towards the targeted delivery of therapeutic agents directly to subcellular organelles, particularly mitochondria, to improve treatment outcomes and minimize systemic side effects. Leveraging subcellular targeting to enhance the therapeutic index presents an enticing strategy, as it enables the direct delivery of therapeutic agents to their intracellular sites of action (1). By specifically targeting subcellular compartments or organelles associated with disease processes, therapeutic molecules can accumulate at the target site in higher concentrations, thereby boosting specificity and efficacy. Targeted delivery methods also permit the administration of lower therapeutic doses, thereby significantly reducing off-target toxicity in non-target tissues and enhancing the overall safety of the treatment strategy (2–4). Furthermore, subcellular targeting supports the real-time monitoring of therapeutic responses at the subcellular level; imaging techniques or the tracking of specific subcellular biomarkers or metabolites can dynamically assess the effectiveness of the treatment. Mitochondria, often described as the powerhouse of the cell, play a crucial role in energy production, apoptosis regulation, and cellular metabolism (5–7). Their central role in various physiological and pathological processes, including neurodegenerative diseases, cancer, and cardiovascular disorders, has positioned them as a strategic target for therapeutic interventions (8, 9). Recent advancements in nanotechnology have enabled the development of nanocarriers designed for the precise delivery of drugs to mitochondria, overcoming biological barriers that have traditionally impeded effective drug distribution at the subcellular level (10–12). These nanocarriers leverage unique physicochemical properties to navigate the complex intracellular environment, ensuring that therapeutic agents are delivered efficiently to mitochondria, thus maximizing therapeutic efficacy while minimizing off-target effects (13). The mitochondria-targeted nanomedicines represents a leap forward in the quest for precision medicine. By focusing on the mitochondria, researchers aim to exploit the organelle’s unique characteristics and vulnerabilities, such as its membrane potential and the presence of specific transporters, to enhance drug accumulation and activity within the target site (14). This approach has shown promise in preclinical studies, demonstrating improved outcomes in models of cancer, mitochondrial diseases, and other conditions where mitochondrial dysfunction plays a key role. As the field continues to evolve, the design and development of mitochondria-targeted nanomedicines are guided by a deeper understanding of mitochondrial biology, the identification of novel targeting ligands, and advancements in nanocarrier technology. This multidisciplinary effort, encompassing the fields of nanotechnology, biochemistry, and pharmacology, is paving the way for the next generation of therapeutics that can selectively modulate mitochondrial functions for therapeutic gain. The strategic targeting of mitochondria using advanced nanomedicine approaches holds the potential to revolutionize the treatment of a wide range of diseases. In the realm of cancer therapy, multidimensional nanoplatforms and systems targeting mitochondria have been developed, significantly enhancing treatment outcomes (15–17). By overcoming the limitations of conventional drug delivery methods, mitochondria-targeted nanomedicines offer a promising platform for enhancing therapeutic outcomes and ushering in a new era of precision medicine. M. Akhtar and colleagues focus on drug delivery systems targeting overexpressed receptors in cancer tissues and the tumor microenvironment in their review. They briefly explore the structure and function of these receptor molecules, highlighting elegant mechanisms that leverage specific characteristics of cancer for therapeutic purposes. Following the discussion on receptors, the review delves into their respective ligands and examines the contribution of nanotechnology to delivering anticancer drugs in preclinical cancer models. Ligand-functionalized nanocarriers have shown significant anticancer drug delivery in various *in vitro* and *in vivo* cancer models compared to cancer models lacking these receptors or nanocarriers without ligand-mediated drug delivery. The enhanced tumor site concentration of anticancer drugs achieved through nanotechnology can significantly impact the efficacy of cancer treatment while minimizing systemic side effects (18).

## 2 Advances in mitochondria-targeted nanotherapy

Mitochondria, the powerhouses within cells, play a crucial role in supporting cellular life activities and metabolic processes (19–21). They are central to critical physiological processes such as electron transport, ATP production, reactive oxygen species (ROS) generation, calcium ion regulation, and the initiation of cell death (22–26). Furthermore, mitochondria are involved in diverse metabolic activities including beta-oxidation of fatty acids, the citric acid cycle, gluconeogenesis, and steroid synthesis, significantly impacting cell fate (27–30). Energy-demanding tissues like the heart, endocrine, visual, and nervous systems are particularly susceptible to mitochondrial dysfunction (31–35). Several approved drugs that exert therapeutic effects by activating apoptosis pathways through direct action on mitochondria underscore the pharmacological importance of mitochondria as targets (36, 37). Targeting mitochondria for drug action presents an effective strategy for addressing cellular metabolic dysregulation and mitochondrial-related diseases (38). Despite the desire to direct therapies specifically at mitochondria, their complexity makes this task challenging.

### 2.1 Mitochondrial structural features and their target potential in disease therapy

**Figure 1:**
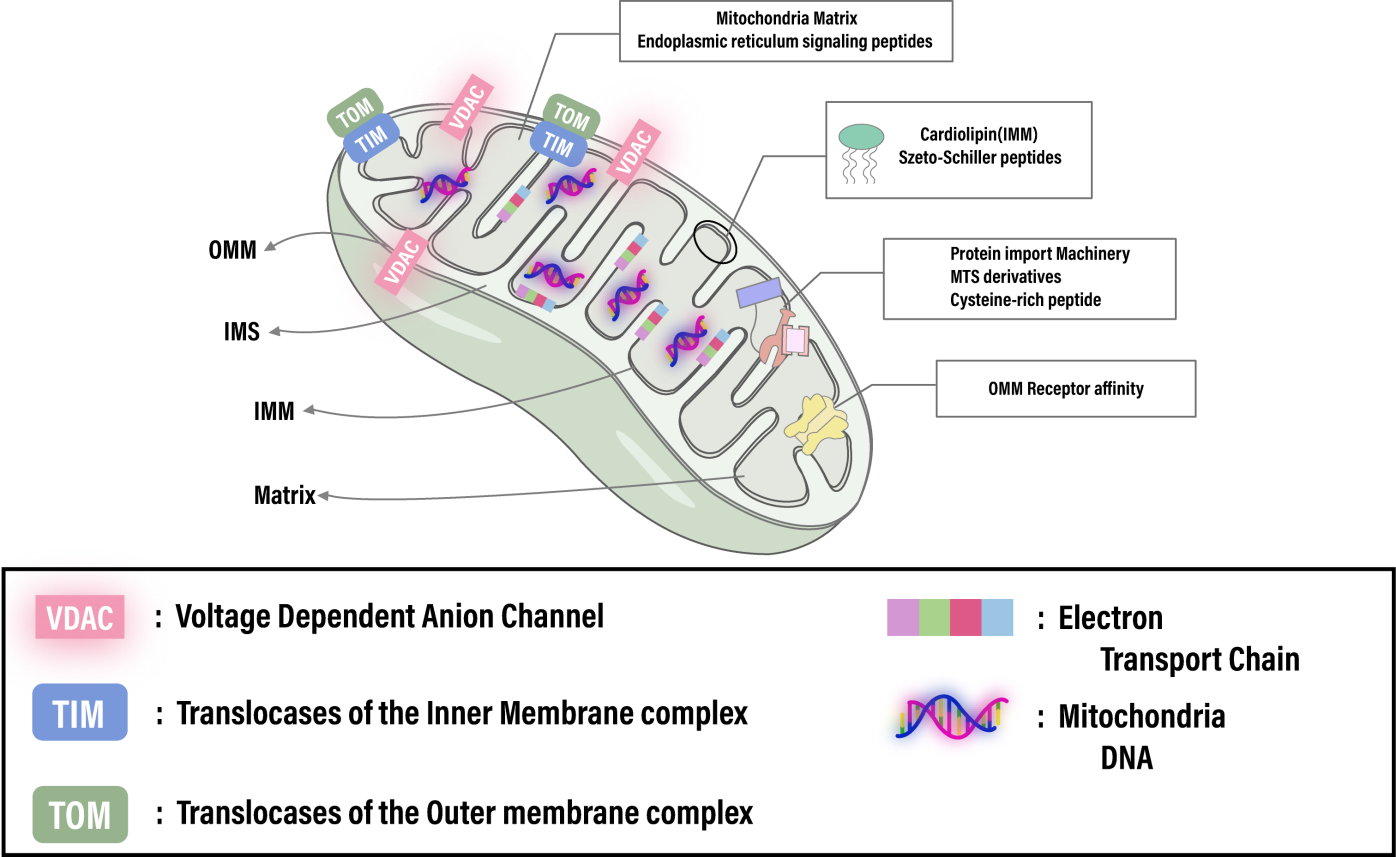
Illustrates the intricate architecture of the mitochondrion, spotlighting crucial elements like the membrane potential, cardiolipin, translocase complexes (TOM/TIM), and receptor affinities essential for targeted therapies. It emphasizes the capability for advanced therapeutic strategies through precise mitochondrial targeting. Adapted from (145).

The uniqueness of mitochondria as organelles lies in their distinctive structural features, making them ideal targets for specific targeting. Composed of two membranes and four compartments—the outer mitochondrial membrane (OMM), the intermembrane space (IMS), the highly lipophilic inner mitochondrial membrane (IMM), and the mitochondrial matrix—mitochondria’s architecture facilitates its functions (**Figure 1**). The OMM is characterized by the voltage-dependent anion channels (VDAC), the most abundant proteins on the outer membrane, allowing small molecules to passively diffuse into the IMS (39). While the OMM is relatively permeable to small molecules, the IMM is almost impermeable to small ions like hydrogen. The IMM’s surface area is increased by folds called cristae, containing numerous proteins involved in the electron transport chain (ETC) and ATP synthesis (40, 41). The ETC, a crucial pathway in mitochondria, involves a series of proton pump complexes (42, 43) that transfer electrons from donors to acceptors through redox reactions, generating energy that creates a proton gradient between the IMS and the matrix, and a strong negative membrane potential. This electrochemical potential energy is then used to drive ATP synthesis. The ETC, along with oxidative phosphorylation and the Krebs cycle, plays a vital role in cellular energy metabolism and ATP production (44, 45). Mitochondria possess their own DNA (mtDNA), yet most mitochondrial proteins and enzymes are encoded in the cell nucleus, translated in the cytoplasm as unfolded precursor proteins (46, 47).

### 2.2 Advancements and applications of nanoparticle targeting to organelles

**Figure 2:**
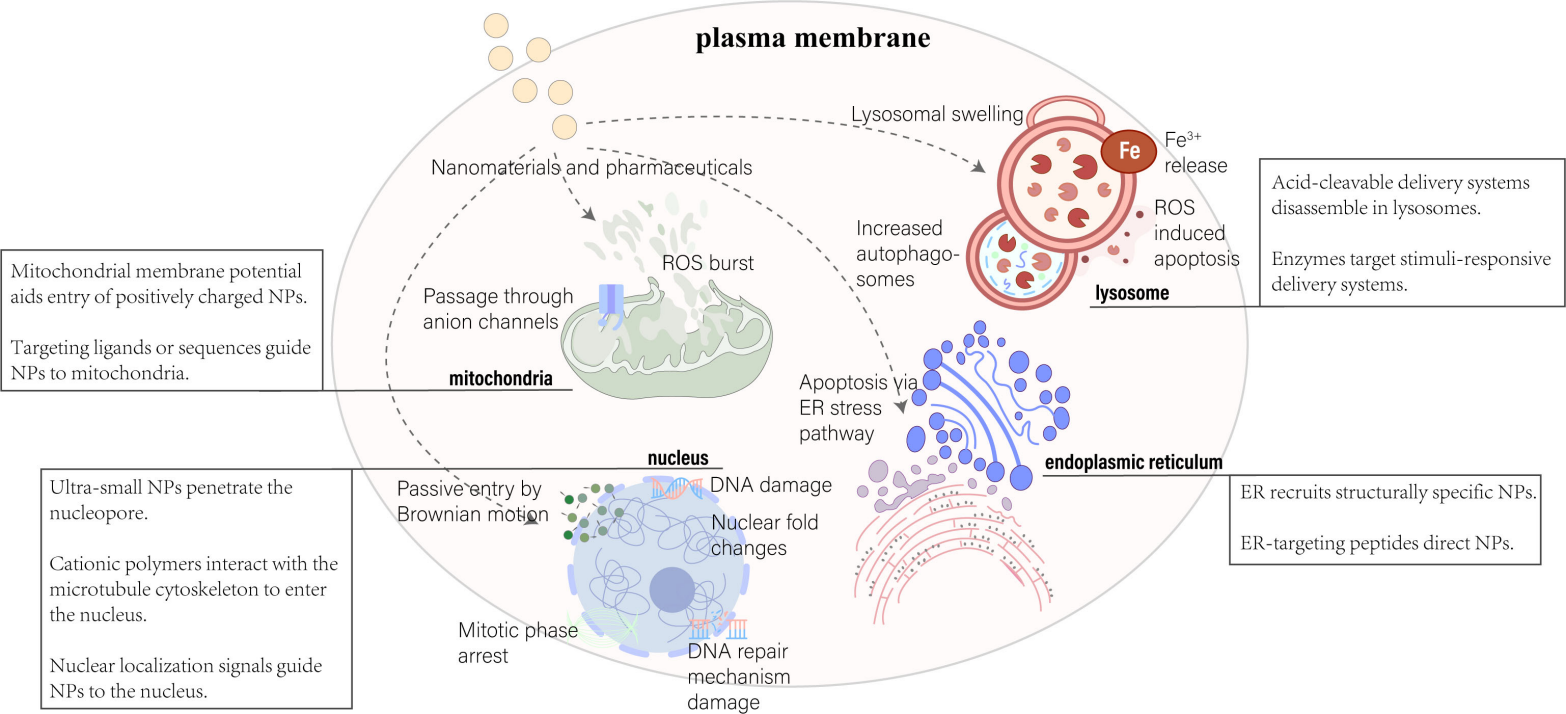
Nanoparticle delivery strategies target key organelles, including the nucleus, mitochondria, lysosome, and endoplasmic reticulum. Various approaches leverage organelle-specific properties and targeting peptides for effective intracellular delivery. Adapted from (169, 170).

Significant advancements in nanotechnology for medical applications have been achieved, from Lipodox in 1995 to the Pfizer-BioNTech COVID-19 vaccine in 2020, marking major breakthroughs in nanomedicine. The development of nanodrugs has enhanced drug accumulation in specific cells through the enhanced permeability and retention (EPR) effect and active targeting techniques for cellular uptake (48). However, despite these advances, the effective concentration of nanodrugs remains low, with studies showing only about 5.6% of injected nanodrugs effectively accumulating at tumor sites (49). Moreover, a meta-analysis found that only 0.7% (median) of the administered nanoparticle dose reaches solid tumors (50). Biological barriers to drug transport hinder the successful accumulation of nanotherapies at diseased sites, limiting effective responses in conditions ranging from cancer to inflammation. Despite extensive research efforts to incorporate multifunctionality and specificity into nanocarrier designs, many strategies fall short of fully overcoming these obstacles. Challenges such as nonspecific distribution and insufficient therapeutic drug accumulation remain significant hurdles for drug developers. There’s a pressing need to reimagine traditional nanoparticle formulations to effectively address these drug delivery barriers (51). Although many factors affect the effective delivery of nanodrugs, understanding physiological conditions in health and disease and rational design can optimize pharmacokinetic properties and modulate the key interactions between nanodrugs and biological barriers that influence their *in vivo* absorption and intracellular/subcellular localization. Third-generation nanodrugs, utilizing subcellular localization strategies, not only optimize therapeutic effects and reduce required dosages but also overcome challenges of peptide and oligonucleotide degradation within cells. These nanocarriers achieve precise delivery to specific organelles by adjusting their chemical properties, size, and shape, thus minimizing organelle toxicity. Key elements in nanodrug design include targeting specific subcellular structures, fine-tuning nanoparticle size, and ensuring charge and biocompatibility to enhance therapeutic efficacy (52) (**Figure 2**). For instance, Zeng et al. developed an innovative ROS-activatable nanoprecursor drug (HTCF) capable of dual-targeting both tumor cells and mitochondria for anticancer therapy. This ingenious organelle-specific nanomedicine demonstrated significant antitumor activity both *in vitro* and *in vivo*, unveiling the potential of amplified tumor-specific oxidative therapies and offering insights for treating various cancers (53).

### 2.3 Biological barriers in drug delivery

**Figure 3:**
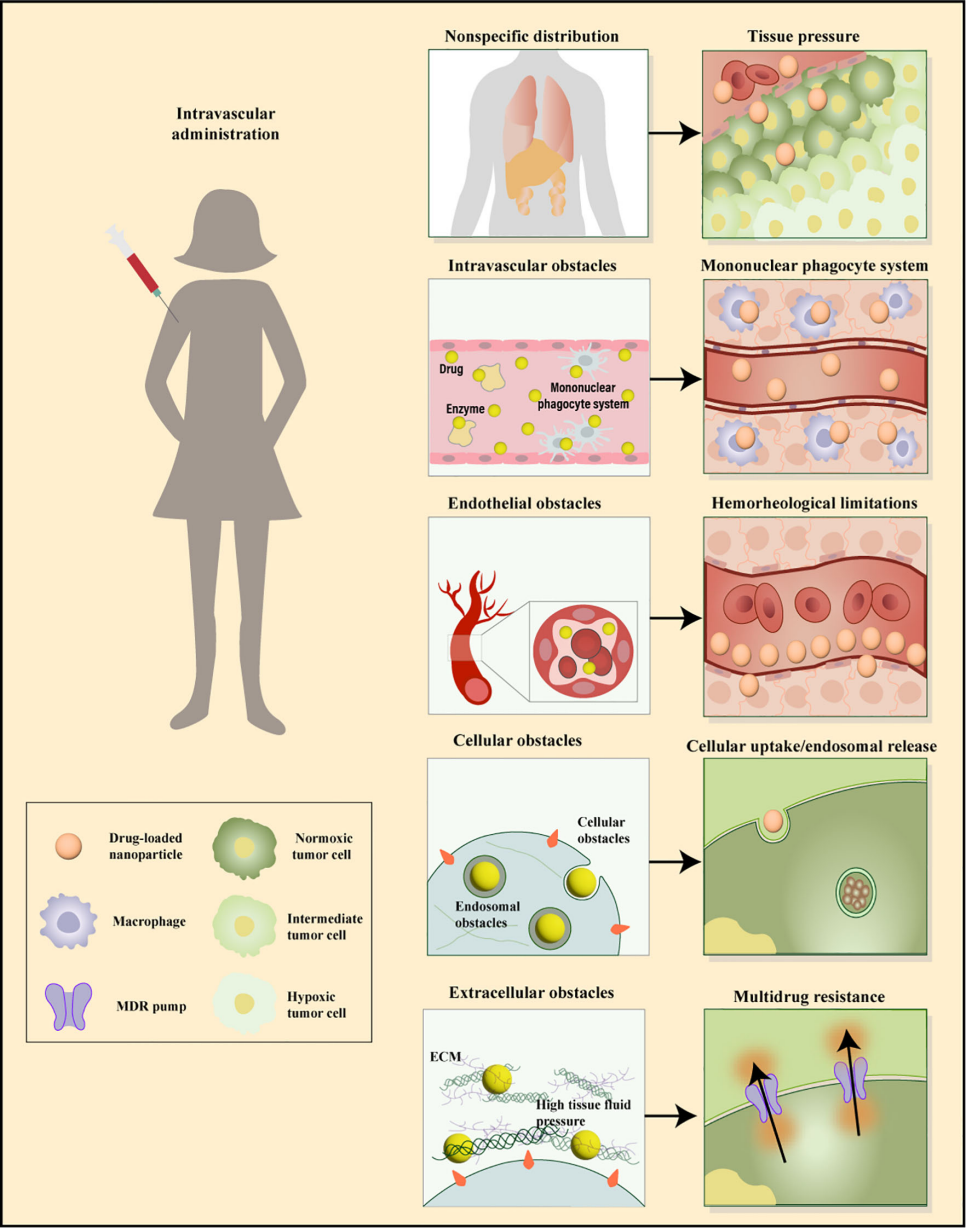
Sequential biological barriers to site-specific drug delivery. After intravenous administration, nanoparticles face multiple obstacles, including opsonization, uptake by macrophages, nonspecific accumulation in organs like the spleen and liver, and challenges in margination dynamics. Additionally, high tissue pressure and cellular barriers such as internalization, endosomal escape, and drug efflux pumps hinder effective targeting and delivery. Adapted from (171, 172).

Nanoparticles (NPs) have emerged as an effective means for treating a variety of diseases, including cancer, cardiovascular, and inflammatory conditions. Various types of NPs have been synthesized, such as liposomes, polymeric particles, micelles, dendrimers, quantum dots, gold NPs, and carbon nanotubes. One of the primary challenges limiting the success of NPs is their ability to reach the therapeutic site in necessary dosages while minimizing accumulation at undesired locations. The biodistribution of NPs is determined by biological barriers in the human body, which manifest in several distinct ways (54). After systemic administration, biological barriers are hierarchically present, including nonspecific circulatory barriers, specific tissue/microenvironment barriers, cellular-level, and subcellular-level barriers (**Figure 3**). The complexity of this classification increases due to the route of administration, target diseases, and interpatient variability, and currently, there is no universal method for effectively overcoming these biological barriers. In designing drug delivery systems to overcome specific barriers, numerous potential parameters are critical. The aim of these systems is to enable drugs to cross barriers that cannot be surmounted with safe doses by conventional means, acting as a modifier of pharmacokinetics (55). Moreover, off-target effects of systemically administered drugs have always posed a significant challenge in designing therapies with intended efficacy and acceptable toxicity. Over the past thirty years, a vast body of literature has focused on understanding biological barriers that impede tissue-specific drug delivery and strategies to overcome these obstacles. This body of work outlines several targeting strategies currently being adjusted in preclinical and clinical settings for drug delivery, including strategies based on small molecules, nucleic acids, peptides, antibodies, and cells (56). However, in addressing these barriers, nanocarriers exhibit significant advantages by enhancing drug solubility, stability, circulation time, targeting, and transcellular barrier release, showing potential applications in oral and tissue delivery (57).

#### 2.3.1 Non-specific/circulatory obstacles

The reticuloendothelial system (RES) pose absorption barriers by recognizing the physicochemical characteristics of nanomedicines, while polyethylene glycol modification (PEGylation) can effectively extend the circulation time of nanomedicines in blood and reduce RES uptake (58, 59). However, PEGylation may decrease the cellular uptake of nanomedicines, impacting therapeutic efficacy (60, 61). Scientists are exploring alternative hydrophilic polymers to PEG, such as poly(ethyleneimine) (PEI), poly(aspartic acid) (PAsp), and sulfated polymers, to mitigate potential complement activation and allergic reactions caused by PEGylated nanomedicines, while also enhancing targeting and reducing immunogenicity (62–64). Moreover, biomimetic camouflage using natural cellular elements (e.g., platelets) and developing non-traditional shapes of nanoparticles (e.g., star-shaped, helical, and polyhedral nanoparticles) are effective strategies to evade the RES system (65, 66). Notably, nanoparticles with irregular shapes enhance their interaction with vascular endothelial cells (e.g., through “lateral migration” or “marginal effect”), thus bypassing the RES and improving drug delivery efficiency. The enhanced permeability and retention (EPR) effect is crucial for enhancing the accumulation of nanomedicines at tumor sites, but its efficiency is influenced by factors such as blood flow, vascularization, interstitial pressure, and vascular obstruction (67, 68). Supplementary mechanisms, like the “hitchhiking” effect of immune cells and active transcytosis by tumor endothelial cells, also contribute to the accumulation of nanomedicines in tumors, overcoming the limitations of the EPR effect.

After discussing the role of PEGylation and alternative hydrophilic polymers in enhancing circulation time and reducing undesired uptake by the reticuloendothelial system (RES), it is crucial to consider another pivotal factor influencing nanoparticle behavior in the bloodstream: the formation of a protein corona. Upon entering the biological milieu, nanoparticles rapidly associate with various proteins in the serum, forming what is referred to as the protein corona (69). This protein layer significantly influences the biodistribution, cellular uptake, and interactions of nanoparticles with target cells (70). For instance, the presence of a protein corona can either enhance or hinder the targeting of nanoparticles to specific cell populations (71). Understanding and controlling the formation of the protein corona is crucial for optimizing nanoparticle-based delivery systems, especially in precise mitochondrial targeting strategies. By finely tuning the surface characteristics and size of nanoparticles, the composition and structure of the protein corona can be manipulated, thereby enhancing therapeutic outcomes and minimizing non-specific interactions (72, 73). This is a pivotal step in advancing mitochondrial-targeted nanotherapeutics and their clinical applications, ensuring higher precision and reduced toxicity in treatments.

#### 2.3.2 Tissue-specific barriers/microenvironment

The uniqueness of blood-tissue barriers and the tumor microenvironment (TME) presents challenges for nanomedicine delivery, yet also offers therapeutic opportunities by leveraging environmental differences. Leveraging unique properties, a dual-triggered activatable cell-penetrating peptide (designated as dtACPP) responsive to lowered extracellular pH and MMP2 was engineered, leading to the successful development of a smart nanoparticle system modified with dtACPP for the dual loading of gene therapies and chemotherapeutics. Upon systemic administration, dtACPP-modified nanoparticles exhibit passive tumor targeting through enhanced permeability and retention effect. Subsequently, dtACPP is activated to expose cell-penetrating peptides, driving the internalization of nanoparticles into tumor cells (74). Leveraging the concept of stimulus-responsive nanomedicine, Chen et al. developed a programmable nanoparticle (NP) delivery system featuring a pH-triggered detachable PEG layer and a reduction-responsive core modified with lactic acid (Lac) to tackle the “PEG dilemma” and enable on-demand intracellular release of doxorubicin (DOX) (75). Similarly, Zhang et al. constructed a pH-responsive polyethylene glycolated hyaluronic acid nanoparticle system (HA-mPEG2k-DOX), which efficiently targets CD44-positive CT26 cells. The pH-responsive cleavable PEG shell dissociates in the mildly acidic tumor microenvironment, facilitating cellular uptake of HA-DOX NPs. This design not only prolongs DOX circulation time and reduces toxicity but also effectively targets CD44-positive tumors, thereby enhancing therapeutic efficacy in colorectal cancer treatment (76).

#### 2.3.3 Cellular and subcellular barriers

The success of engineered nanomedicines hinges on their intracellular and subcellular localization within the target environment, with internalization pathways and subcellular targeting being critical to their therapeutic effectiveness (77, 78). Optimized design allows nanomedicines to enhance therapeutic outcomes, reduce required dosages, and minimize off-target effects. Design considerations must include endosomal escape, specific organelle characteristics, and uptake efficiency to ensure precise and efficient drug delivery. A reported nanocarrier system, DF-MTS-MITO-Porter, has been developed for mitochondrial delivery to modulate intricate intracellular processes. This system is designed not just for the delivery of small molecule compounds but also for transporting proteins/nucleic acids to regulate mitochondrial functions (79).

### 2.4 Strategies for overcoming mitochondrial biological barriers

Effectively delivering specific drugs to mitochondria requires not only solving intracellular transport issues but also precisely crossing the mitochondrial outer and inner membrane barriers. The membrane potential difference of mitochondria is a key factor for drug entry into its matrix, with lipophilic and positively charged molecules able to accumulate in mitochondria by exploiting this potential difference (80). The design of multifunctional nanocarriers targeting mitochondria has become a research focus in recent years. These nanocarriers can bypass *in vivo* barriers, increase bioavailability, and enable controlled release of therapeutic agents at target sites, facilitating synergistic effects in treatments like chemotherapy, gene therapy, and phototherapy (81). An acid-activated mitochondrial-targeting drug nanocarrier has been developed for precise delivery of nitric oxide (NO) as an ATP synthase inhibitor, showing extended blood circulation, enhanced cellular uptake, and restored mitochondrial targeting ability under the extracellular pH value (6.5) of tumor cells (82). The transport of bioactive molecules to mitochondria primarily depends on the mitochondrial membrane potential and specific protein absorption mechanisms. Scientists have found that specific mitochondrial targeting molecules, especially amphiphilic cations, can recognize and target the mitochondrial membrane potential, thereby facilitating drug delivery to mitochondria. For example, acid-activated nanoparticles developed by Qi et al. initially target endosomes before migrating to mitochondria, significantly enhancing therapeutic outcomes by triggering fluorescence enhancement and photosensitization, inducing cancer cell death upon laser irradiation (83). Triphenylphosphonium (TPP) moieties are widely used in mitochondrial targeting for their ability to promote selective accumulation of bioactive molecules. In Liang et al.’s study, a core-shell structured upconversion nanocrystals-dendrimer complex targeting mitochondria and overcoming tumor hypoxia enhanced the efficacy of anticancer photodynamic therapy; catalytic degradation of hydrogen peroxide (H2O2) by catalase overcame tumor hypoxia and mitochondrial targeting, significantly enhancing PDT efficacy and offering a new paradigm for cargo delivery (84). Sun et al. reported a mitochondrial-targeting nanocarrier designed with poly(lactic-co-glycolic acid) (PLGA) and TPP, which significantly increased levels of interferon-gamma (IFN-γ) activated by mitochondrial-targeting immunotherapy in cancer cells. These TPP-modified mitochondrial-targeting polymer nanoparticles can be used for cancer treatment, substantially improving therapeutic outcomes (85).

Another strategy for mitochondrial targeting involves using mitochondrial targeting ligands or mitochondrial targeting sequences (MTS). For example, Lopez and their team developed Janus mesoporous silica particles asymmetrically decorated with two targeting moieties: one selective for folate membrane cell receptors (folate) and the other capable of binding to the mitochondrial membrane (triphenylphosphonium, TPP), facilitating sequential vectorization from the cell to the organelle. Compared to symmetric nanocarriers, the asymmetric decoration on each side of the particles allows for precise control during the targeting attachment process. The presence of folate induces increased particle accumulation inside tumor cells, where these nanocarriers are then guided to the vicinity of mitochondria through the action of the TPP moiety. This strategy enhances the therapeutic effects of current nanomedicines (86). Qi and colleagues explored using a specific protein, mitochondrial targeting signal protein (MLSP), for targeting mitochondria. The MLSP-modified polymer nanoparticles they developed were efficiently taken up by cells and localized to mitochondria, demonstrating potential for direct intervention in mitochondrial dynamics (83). Most mitochondrial targeting strategies utilize mitochondrial signal sequences to direct complexes to mitochondria, and recognizing and exploiting this mechanism lays the foundation for new therapeutic developments. Gao and others effectively achieved precise mitochondrial localization by integrating MTS sequences on the surface of biocompatible nanocarriers, triggering significant antitumor activity in cancer cells (87). Moreover, researchers have been able to more precisely localize therapeutic agents to mitochondria, enhancing therapeutic efficiency and reducing impacts on normal cells, by utilizing hybrid peptide systems with specific peptide sequences. This approach, by disrupting mitochondrial membrane stability, directly affects mitochondrial function, paving new pathways for cancer treatment (88). The development and application of mitochondrial penetrating peptides (MPPs) showcase the great potential of directing nanoparticles to mitochondria through specific sequences. The design and optimization of MPPs are expected to play a significant role in future mitochondrial targeting strategies, offering new directions for treating mitochondrial-related diseases (89).

### 2.5 Comparative effectiveness of mitochondrial targeting sequences

It is imperative to assess the unique mechanisms and effectiveness of Mitochondrial Targeting Sequences (MTS), Mitochondrial Processing Peptidases, and Mitochondrial Localization Signal Peptides (MLSP). These sequences each play a crucial role in directing nanomedicines to mitochondria, distinguished by their distinct interactions with mitochondrial membranes and import machinery. Typically positioned at the N-terminus of proteins, MTSs are cleaved upon entry into mitochondria and directly interact with the translocase of the outer membrane (TOM) complex (90). This interaction facilitates the entry of nanocarriers or therapeutic agents into the mitochondrial matrix. The effectiveness of MTSs is shaped by their amino acid composition and structural configuration, influencing their recognition and processing by mitochondrial import machinery. Although not a targeting sequence itself, Mitochondrial Processing Peptidases is pivotal in the maturation of proteins transported into mitochondria by cleaving their targeting sequences (91). This enzymatic action is vital for the functionality of MTS-containing proteins, ensuring they are correctly processed and activated within mitochondria. The efficiency of this process is influenced by the sequence and context of the cleavage site, which can significantly impact the overall success of the mitochondrial targeting strategy. MLSPs are instrumental in the precise localization of proteins to specific mitochondrial compartments—such as the intermembrane space, inner membrane, or matrix. The specificity and efficiency of MLSPs are vital in scenarios where the exact sub-mitochondrial positioning of a therapeutic agent is crucial for its efficacy. Studies demonstrate that the success of these targeting sequences varies based on the type of nanoparticle, the therapeutic agent used, and the specific mitochondrial function targeted. While MTS is generally preferred for its widespread applicability and effectiveness in delivering agents to the mitochondrial matrix—a site of numerous vital metabolic processes-MLSP may be better suited for targeting specific compartments within mitochondria that are essential for activating apoptotic pathways in cancer therapies. Further research is essential to conclusively determine the relative effectiveness of these sequences as their performance may also be contingent on the physicochemical properties of the nanocarrier and the pathological context of the treatment. Continued studies and clinical trials are expected to enhance our understanding of how to best utilize these mitochondrial targeting strategies, paving the way for more refined and effective therapeutic approaches.

## 3 Nanomedicines targeting mitochondria

**Figure 4:**
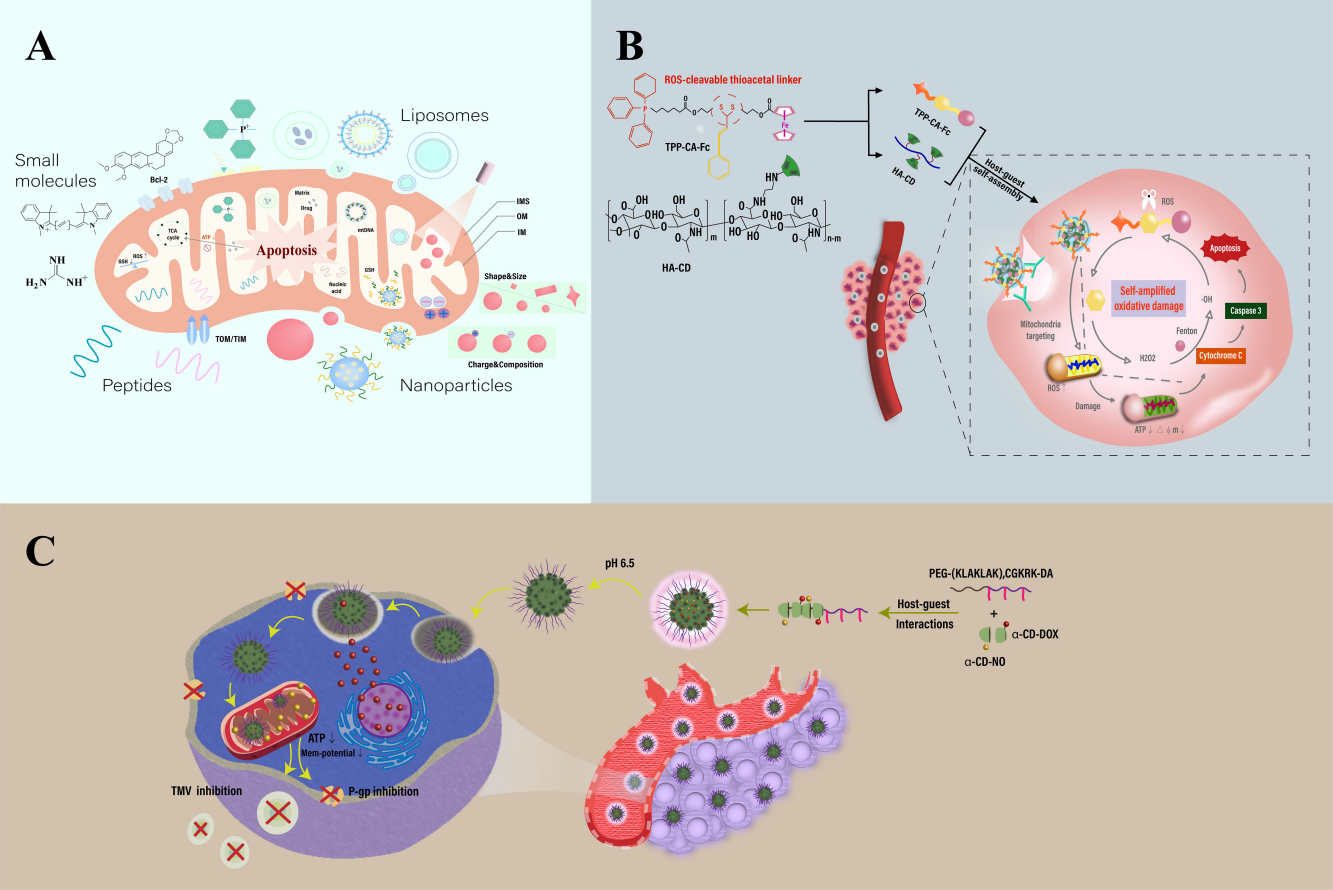
Schematic illustration of mitochondria-targeting-based nanotechnology. **(A)** Construction and therapeutic strategies of mitochondria-targeted nanosystems for therapy. **(B)** Overview of a mitochondria-targeted, ROS-activated nanomedicine for cancer therapy. Features a ROS-sensitive thioketal linker (TK) for targeted drug release within mitochondria. The structure consists of self-assembled α-cyclodextrin (α-CD) and hyaluronic acid (HA) conjugates linked to a ROS-responsive prodrug via TK. It also incorporates triphenylphosphine (TPP) and Fenton-catalyzed ferrocene (Fc), enhancing mitochondrial oxidative damage and promoting cell apoptosis through chemodynamic therapy. **(C)** Overview of a cyclodextrin-based nanomedicine designed for targeting mitochondria in cancer therapy, utilizing mitochondrial membrane potential (MMP) and charge-reversal techniques. The diagram outlines the composition and delivery mechanism of α-cyclodextrin (α-CD) nanoparticles. These particles are equipped with nitric oxide (NO, attached via GSH) and doxorubicin (DOX, linked through hydrazine), and are further enhanced with dopamine-conjugated PEG attached to a mitochondrial penetrating peptide sequence, (KLAKLAK)2CGKRK. This configuration boosts the treatment’s targeting precision and effectiveness. Adapted from (53, 173, 174).

Murphy’s team developed a series of mitochondria-targeted antioxidants that are specifically absorbed by mitochondria through covalent linkage with lipophilic cations such as triphenylphosphonium (TPP) (92). Besides TPP and rhodamine, certain specialized lipophilic cations have also been utilized for mitochondria-targeted delivery research (93). By combining these lipophilic cations with antioxidant molecules, Murphy’s team developed targeted drug delivery systems for mitochondria. Although these compounds are cost-effective, stable, and can be conveniently chemically conjugated with nanocarriers for mitochondrial targeting, their toxicity at high concentrations, especially in scenarios that might disrupt mitochondrial membrane potential, limits their application. Meanwhile, Zielonka and colleagues introduced a new category of mitochondrial targeting compounds known as delocalized lipophilic cations (DLCs), which have gained widespread attention due to their direct absorption by mitochondria. Zielonka’s research indicates that DLCs, through their unique structure, serve as significant targets in mitochondria for drug design in cancer, cardiovascular diseases, and neurodegenerative diseases (93). Designing mitochondria-targeted nanomedicines involves considering strategies to cross multiple biological barriers in the body, including optimizing the bioavailability and distribution of the drug. Nanocarrier design emphasizes biocompatibility, degradability, size, lipophilicity, charge, and the selection of targeting elements to ensure precise drug release (**Table 1**), (**Figure 4**). Multifunctional nanocarriers use materials like liposomes, polymer nanocarriers, and mesoporous silica, focusing on their biocompatibility and biodegradability. By incorporating mitochondrial targeting peptides or DLCs and using polyethylene glycol (PEG) to enhance blood stability, these carriers improve mitochondrial localization and therapeutic efficiency. For example, lipid polymer hybrid nanoparticles (LPNPs) consisting of poly(D,L-lactide-co-glycolide) (PLGA), TPP-containing amphiphilic polymers (C_18_-PEG_2000_-TPP), and reduction-responsive amphiphilic polymers (DLPE-S-S-mPEG_4000_), after detaching the PEG through redox reactions, achieved precise and rapid localization and exhibited high anticancer activity (94).

**Table 1:** Examples of mitochondria-targeted organic nanomedicines demonstrating specific trigger-responsive drug release, which have reached preclinical testing for various applications.

Type of encapsulate	Nanomedicine	Disease	Material	Formulated Drug/Bioactive	Targeting Moiety	Targeting Mechanism	Strategy characteristic	Preclinical model	Ref.
Polymeric-based									
**micelles**	α-CD-DOX-NO-DA nanoparticles	Breast cancer	α-CD nanocarrier with an acid-cleavable dimethylmaleic anhydride (DA) moietymodified PEG conjugated to an MPP (KLAKLAK)2CGKRK	DOX (hydrazone linker) NO (disulfide bond)	(KLAKLAK)2CGKRK (MPP) (mitochondria targeting)	Charge-reversal NO membrane permeabilization MPP helps target mitochondria	charge reversal (acid-cleavable DA moiety) GSH-triggered NO release	4 T1 and MCF7 mice models	(77)
	TDTD@UA/HA	Cancer multidrug resistance (MDR)	TPP-DEX-TK-DOX conjugate	DOX(amide bond and conjugated) UA(encapsulated)	TPP (DLC) (mitochondria targeting) HA (CD44 bind) (cellular targeting)	HA ligand-CD44 receptor interaction degradation of HA layer TPP helps target mitochondria self-boosted release of UA and DOX	cascade targeting charge reversal(HA removed) thioketal linker(TK) (ROS sensitive)	MCF-7/ADR cells and MCF-7/ADR tumor-bearing nude mice	(155)
	CS-g-SS-31/PDAA@PU	Ischemia-reperfusion injury	conjugate Chitosan-SS-31 via ROS-sensitive thioketal (PDAA)	PU (Puerarin) (thioketal crosslinking)/SS-31	SS-31 (mitochondria targeting)	intracellular transport SS-31 peptide helps target mitochondria	Intranasal admin (ischemic penumbra) bypassing the blood−brain barrier thioketal linker(TK) (ROS sensitive)	SH-SY5Y /middle cerebral artery occlusion rats	(156)
	G3-C12-HPMA-KLA	Cancer	HPMA conjugated to KLA (D(KLAKLAK)2) and G3-C12 peptide (ANTPCGPYTHDCPVKR)	KLA peptide	G3-C12 peptide galectin-3-targeting ligand (cellular targeting)(mitochondria targeting)	G3-C12 peptide ligand helps target mitochondria and cellular	cellular-mitochondria dual targeting(G3-C12 peptide)	PC-3 cells and tumor-bearing nude mice	(157)
	CS/TPP-PEG-ss-PLA@DOX	Hepatocellular carcinoma	TPP grafted PEG-ss-PLAcopolymers coated with CS	DOX (encapsulated)	CS (CD44 receptor affinity) (cellular targeting) TPP (mitochondria targeting)	CD44-mediated endocytosis pH response( CS is removed,exposing the positively charged TPP) DOX released due to GSH-triggered disassembly.	pH/redox dual response cascade targeting covered by CS layer	HepG2 cells and H22 bearing Balb/c mice	(158)
	PEGm-Fn	Cancer	PEG-core-fluorinated micelles with fluorocarbon	vitamin E succinate (VES) Fluorescent dyes (encapsulated)	Fluoroamphiphiles (mitochondria targeting) strong binding affinity with the phospholipids low cytotoxicity	Clathrin-dependent endocytosis and endosome escape Fn helps target mitochondria by cardiolipin affinity	charge-neutral low cytotoxicity strong binding affinity with phospholipids	HeLa, ovarian NCI/ADR-RES, and breast cancer cell lines/orthotopic 4 T1 mouse model	(138)
	Cyt C-encapsulated CD NPs, Co-delivery of CQ	Cancer	cationic dextrin,cationized by glycidyltrimethylammonium chloride (GTMAC), sodium tripolyphosphate (TPP)	Cyt-C(encapsulated)	GTMAC(mitochondria targeting)	GTMAC helps target mitochondria chloroquine (CQ) help enhance endosome escape Endosomal rupture, membrane depolarisation mediated by Cyt-C	biodegradability	HeLa cell, A549 cells	(159)
	NPs@Cur	Acute Kidney Injury(AKI)	PEG-b-PAGA/ PDMAEMA-r-PAAPBA)-b-PPBAE/Cur	Cur (encapsulated)	N/A	Endocytosis and endosomal escape after protonation (PDMAEMA), PAGA and PAAPBA form cross-linked boronic esters ROS-induced degradation of NPs	Borate esters (ROS sensitive) dual-response based on dynamic covalent chemistry	tubular renal HK-2 cells/AKI mice, yes/no cisplatin treatment	(160)
	TPP11–malonate	Cancer	TPP -dicarboxylate malonate	Malonate/TPP	TPP (mitochondria targeting)	TPP helps target mitochondria	cleaved by intramitochondrial esterase activity uptaken depending on the pH gradient	C2C12 mouse myoblasts and HeLa cells/C57BL/6J mice	(161)
**Dendrimers**	Glucose-PEG–Peptide–Triphenylphosponium–PAMAM–PTX Conjugate	Breast cancer	PAMAM dendrimer-based conjugate derivatized with glucose-PEGGPLGIAGQ (MMP2-degradable peptide)	PTX (disulfide bind)	Glucose-PEG (GLUT1 receptoraffinity) (cellular targeting) TPP (mitochondria targeting)	active endocytosis mediated by GLUT1 MMP2 expression detaches PEG layer TPP helps target mitochondria PTX released by GSH-disulfide linker	cascade chemotherapy overcome multidrug resistance in cancer	MCF7 cells and MCF7 tumor-bearing mice	(148)
	the protein-DNA conjugate consisting of PMK and G-XQ-2d,and loaded with DOX	Multidrug resistance (MDR)	dendrimer-like DNA G modified with aptamer XQ-2d(G-XQ-2d)	anticancer peptide KLAKLAKKLAKLAK (KLA) DOX	KLA(mitochondria targeting) MMP2(cellular targeting)	KLA helps target mitochondria CD71-mediated endocytosis	synergistic peptide-chemotherapy EPR effect helps enhanced stability and passive tumor targetability	MCF-7/ADR, MCF-7, MDA-MB-231, A549 and L02 cells	(162)
**polymersomes**	IrPS+si-OGG1@ABBPN	Glioblastoma multiforme (GBM)	ApoE-mediated blood−brain barrier-penetrating nanocarrier ABBPN	IrPSsi-OGG1(encapsulated)	P-selectin (CD44 bind) (cellular targeting) ApoE(cellular targeting)	active endocytosis by LDLRs P-selectin ligand-CD44 receptor interaction photodynamic ablation (Permeable for IrPS)	cell membrane cloaking process targeted photodynamic therapy alter mitochondrial electron flow	GL261-cells and GBM tumor-bearing mice	(163)
Lipid-based									
**Lipid Nanoparticles**	MTP/RGD-CAL/TAN NS	myocardial infarction	DSPE-PEG-COOH, injectable soya lecithin, PLGA, SS-31, cRGD-PEG-DSPE	Calycosin and Tanshinone (encapsulated)	cRGD for αvβ3 integrin receptor targeting (cellular targeting) SS-31 (mitochondria targeting)	Endocytosis mediated by cRGD/integrin receptor mitochondria targeting by SS-31	co-modified nano-system	Human cardiac myocytes (HCM) acute myocardial infarction in rats	(164)
	PLGA/CPEGT/DSSP@PTX	Breast cancer	Lipid shell: (C18-PEG2k-TPP, CPEGT) (DLPE-SS-mPEG4K, DSSP) Polymeric core: PLGA	PTX (encapsulated)	TPP (mitochondria targeting)	breakage of a pH-sensitive hydrazone linker GSH cleaves the disulfide bonds between POSS and HPMA polymer mitochondria targeting by TPP-Dox	pH sensitive POSS cage tshields of the positive charge Anti-Metastasis Activity by TD	MCF7 cells/MCF7 tumor-bearing mice	(151)
**liposomes**	SMAC-P-FRRG-DOX encapsulated PEGylated liposomes	Multidrug resistance (MDR)	PEGylated liposomes (Aposomes) cathepsin B-cleavable peptide (FRRG)	SMAC-P: AVPIAQ,DOX(FRRG peptide bind)	N/A	EPR effect helps target tumor tissues prodrug is cleaved into SMAC-P and DOX by cathepsin B overexpressed in cancer cells	EPR effect helps enhanced stability and passive tumor targetability special sensitize by IAPs and SMAC-P improve the effect of DOX,inducing a high ICD	CT26 and 4T1 cancer cells	(165)

Adapted from (82, 94, 168, 174, 181–191).

### 3.1 Polymer nanomedicines

Polymer nanomedicines bring multifunctionality to mitochondrial-targeted therapies through their biodegradability, functional diversity, and stimulus-responsiveness. Surface modification of polymer nanocarriers with DLCs (delocalized lipophilic cations) and MPPs (mitochondrial penetrating peptides) endows them with mitochondrial targeting capabilities, creating novel mitochondrial-targeted drug delivery systems (95, 96). Challenges include the potential toxicity of DLCs, stability issues of MPPs, and complex design requirements. To address these issues, research into new targeting ligands and polymers with mitochondrial affinity is underway. Innovative nanocarrier designs include charge-reversal materials that enhance intracellular uptake through charge changes under specific conditions and self-assembling micelles that show enhanced anticancer effects through charge conversion. A novel nanoparticle system integrates chemotherapeutic agents with mitochondrial ROS inducers, leveraging pH sensitivity and GSH (glutathione) depletion for improved tumor targeting and efficacy. This nanocarrier uses pH-responsive materials that alter conformation in acidic tumor environments, facilitating drug release and uptake (97). For instance, Deng and colleagues developed an α-cyclodextrin (α-CD) nanocarrier combining chemotherapeutic drugs, mitochondrial ROS inducers, and nitric oxide (NO) donors. Through pH-sensitive and GSH-triggered mechanisms, it achieves tumor-specific drug release, significantly enhancing antitumor activity against a drug-resistant breast cancer model. It promotes mitochondrial membrane permeabilization, reduces ATP levels, and enhances the uptake and retention of DOX, helping to overcome drug resistance and inhibit cancer metastasis (82). Wu et al. reported ROS and GSH-responsive nanoparticles for delivering NO prodrug, such as S-nitrosoglutathione (GSNO), chemically coupled to amphiphilic block copolymers, demonstrating high NO load capacity, stability, and sustained NO release with specific GSH-activated kinetics. These GSNO-functionalized nanoparticles, triggering doxorubicin (DOX) delivery in an ROS-activated manner, enhanced intracellular DOX accumulation while exhibiting good biocompatibility in normal healthy cells due to physiological ROS concentrations. Their work showcases the potential of multifunctional nanoparticles as an effective platform for co-delivering NO and DOX, selectively killing chemotherapy-resistant cancer cells by increasing chemotherapy sensitivity (98). Additionally, Wang et al. demonstrated the advantages of targeting cancer cell mitochondria against resistance, significantly enhancing paclitaxel(PTX) mitochondrial delivery efficacy with paclitaxel (PTX)-loaded TPH(TPH/PTX) nanomicelles. This approach promotes mitochondrial targeting with positively charged TPP medium to overcome lung cancer cell resistance, illustrating the therapeutic potential of mitochondrial targeting delivery in treating various resistant cancers (99). Reports indicate that chemoresistant cancer cells adapt to intrinsic oxidative stress by upregulating their antioxidant systems, leading to increased intracellular GSH content. Doxorubicin, one of the most widely used drugs in tumor treatment, kills cancer cells through various mechanisms but its use is limited by toxicity and chemoresistance. Thus, new therapeutic strategies that reduce dosage and overcome chemoresistance are needed. Daga, M, et al. developed novel glutathione-responsive cyclodextrin nanosponges (GSH-NS) that preferentially release anticancer drugs in cells with high GSH content. In cancer cells with high GSH, doxorubicin-loaded GSH-NS inhibited clone growth, cell viability, topoisomerase II activity, and induced DNA damage, showing higher efficacy than the free drug. Moreover, GSH-NS reduced human tumor development in xenograft models compared to the free drug, suggesting GSH-NS as a suitable drug delivery carrier for future cancer therapy applications (100). However, the charge-reversal strategy faces challenges of low charge conversion efficiency and dependency on heterogeneous stimuli, requiring careful consideration of off-target toxicity.

### 3.2 Lipid-based nanosystems

Lipid-based materials are ideal for mitochondrial-targeted nanomedicines due to their biocompatibility, simple formulation, self-assembling capabilities, and potential to form hybrid nanomedicines with polymer materials. Membrane fusion technology, an effective mitochondrial targeting strategy, utilizes special fusion lipids to integrate endosomal and mitochondrial membranes. Yamada and colleagues developed the MITO-Porter system, which employs lipid derivatives combined with mitochondrial targeting signal peptides for direct mitochondrial delivery through specific membrane fusion, effectively avoiding lysosomal degradation. Enhanced by MTS, this approach improves delivery efficiency and reduces toxicity. The optimized MITO-Porter has shown significant antitumor activity in drug-resistant kidney cancer models and photodynamic therapy. Further optimization of the MITO-Porter system involves adjusting lipid composition and surface functionalization strategies for more efficient and safe mitochondrial targeting (101). DQAsomes, vesicles self-assembled from dequalinium, target mitochondria for gene and chemotherapy, facing challenges such as low efficiency and toxicity (102). However, recent advancements, including pH-sensitive, PEGylated versions, show promise for clinical translation with improved performance and reduced side effects. Lipid-polymer hybrid nanoparticles, combining a polymer core, lipid layer, and PEGylated surface, overcome limitations of frontier mitochondrial targeting nanomedicines like DQAsomes and Mito-Porters, such as low encapsulation efficiency and instability. Moreover, Félix Sauvage and colleagues explored polyethylene glycol-coated liposomes suitable for drug delivery, observing greater stability when prepared in water compared to phosphate-buffered saline. This highlighted the role of electrostatic interactions between the positive charge on DQ and the polar head groups of lipids (103). They exhibit excellent drug release and mitochondrial localization capabilities, offering new strategies for cancer treatment.

### 3.3 Mitochondria-targeting peptides

Szeto-Schiller (SS) peptides possess the ability to penetrate cell membranes, selectively accumulate in the inner mitochondrial membrane (IMM), and scavenge reactive oxygen species (ROS) (104). These short tetrapeptides, composed of alternating aromatic and basic amino acids, carry a positive charge that facilitates free cellular penetration at physiological pH. The mitochondrial targeting mechanism of SS peptides is not fully understood, but it is speculated that their net positive charge promotes attraction to the negatively charged phospholipids in the IMM. Antioxidant peptides such as SS-01, SS-02, and SS-31 have demonstrated significant efficacy, with SS-31 has been used to study protective effects in type 2 diabetic (105). Mitochondrial penetrating peptides achieve efficient intracellular uptake and specific mitochondrial localization through the inclusion of cationic and highly hydrophobic residues (106). MPPs are notable for their tunability and ease of synthesis, making them suitable for various cellular and *in vivo* applications. Mitochondrial targeting sequence peptides are recognized by mitochondrial surface receptors and consist of longer amino acid chains, and also promotes translocation into mitochondria by interacting with the membrane potential present on the inner mitochondrial membrane (107). MTS peptides have succeeded in delivering proteins and nucleic acids but face challenges due to their large molecular size, poor solubility, and cell membrane permeability issues. While mitochondrial targeting peptides hold potential for drug delivery, limitations maybe include restrictions on the size and type of cargoes and the inability to access specific mitochondrial compartments (108). Developing customizable multifunctional delivery systems to address specific mitochondrial delivery needs represents a future research direction.

### 3.4 Mitochondria-specific stimuli-triggered nanomedicine release

Mitochondrial-specific features can enhance the accumulation and targeting of nanomedicines, allowing for the specific release of therapeutic cargoes at the mitochondrial level in response to particular stimuli (**Table 2**). A key strategy involves designing mitochondrial ROS-responsive nanomedicines utilizing TK linkers, biodegradable thioether derivatives that cleave in ROS environments. Yu and colleagues developed a ROS-responsive nanomedicine delivery system that combines mitochondria-targeted cerium oxide nanoparticles with atorvastatin for the treatment of acute kidney injury (AKI). This system demonstrates significant targeting and enhanced cellular uptake. By targeting elevated ROS in AKI, it releases drugs in ROS-rich environments, mitigating excessive ROS through mitochondrial targeting. This reduces oxidative stress and inflammation, effectively protecting renal structures and decreasing tubular cell apoptosis and necrosis. The system not only improves antioxidant and anti-apoptotic effects but also showcases its therapeutic potential in a sepsis-induced AKI mouse model (109). Research by Plotnikov et al. revealed the protective effects of the mitochondrial-targeted antioxidant SkQR1 on the kidneys of neonatal rats in an LPS-induced AKI model. The study confirmed SkQR1 could reduce urine expression of AKI marker neutrophil gelatinase-associated lipocalin (NGAL) and decrease blood urea and creatinine levels, effectively protecting mitochondrial structures from damage (110). SS-31, a mitochondria-targeted peptide known for its potent antioxidant activity, is an excellent candidate for treating acute kidney injury. Addressing existing limitations, Liu et al. developed pH-responsive and AKI renal-targeted nanopolysomes (NPs) that efficiently deliver SS-31. In the acidic lysosomal environment, the electrostatic equilibrium of these NPs is disrupted, facilitating the release of SS-31 and its subsequent targeting to the mitochondria for therapeutic effects. These NPs have demonstrated remarkable capabilities in reducing oxidative stress, protecting mitochondrial integrity, diminishing inflammatory responses, and decreasing apoptosis and necrosis in renal tubular cells following intravenous administration (111). New nanotechnology-based therapies have shown considerable potential in treating acute kidney injury (AKI). Xu and others developed a nanoparticle platform for anti-tumor therapy specifically targeting the mitochondria of breast cancer. This strategy utilized bovine serum albumin (BSA) nanoparticles, modified with tumor-targeting aptamers and combined with a mitochondria-targeted ROS-activated prodrug. Utilizing the Fenton reaction to convert H2O2 into highly cytotoxic hydroxyl radicals, this nanoparticle platform targeted mitochondria within tumor cells. ROS-induced degradation of the TK linker released active components, promoting H2O2 production, enhancing cytotoxicity, and triggering mitochondria-mediated apoptosis. In a breast cancer mouse model, this nanoparticle platform significantly inhibited tumor growth, showcasing the potential of mitochondrial-targeted chemodynamic therapy (112).

**Table 2:** Methods used in studies of mitochondria-targeted nanomedicine to assess targeting efficacy and induced changes.

Technique	Type of analysis	Strengths	Limitations	Application in relevant studies
**Flow cytometry**	ROS levels ATP levels MMP	Individual cell analysis Precise analysis Non-destructive technique High throughput	Dyes required Requires initial visual evaluationExpertise required Expensive and slow	(158)
**Seahorse XF Analyzer**	Mitochondrial respiration Metabolic alterations	obtain OCR and ECAR information Assessment of metabolic alterations using specific kits Tissue measurements possible Non-destructive technique	accuracy depends on Cell culture conditions and confluence time consuming and expensive Normalization to cell number required	(138)
**Western Blotting**	Mitochondrial targeting Mitochondrial protein expression	Widely available method Selectivity for the desired protein technological maturity	isolation requirment only protein cargo can be assessed time-consuming Destructive technique	(166)
**HPLC-UV**	Mitochondrial targeting	Precise and reproducible Rapid analysis and automation Non-destructive technique	professional and Expensive equipment isolation requirment	(151)
**Fluorescence spectroscopy**	Mitochondrial targeting ROS levels ATP levels MMP	simple and rapid analysis Highly selective and sensitive Non-destructive technique	isolation requirment Introduction of artifacts when using fluorophores autofluorescence is not allowed Environmental sensitivity	(156)
microscopy				
**confocal laser scanning microscopy(CLSM)**	Mitochondrial targeting Morphological alterations MMP ROS levels	simple experimental protocol 3D live imaging Detects spectral changes of a fluorescent dye (FRET and BRET phenomena) Non-destructive technique dynamic tri-dimensional tracking	relatively low resolution marker binding may fail due to molecular interactions indirect fluorophore-mediated detection	(158)
**Transmission electron microscopy(TEM)**	Mitochondrial targeting Morphological alterations	unique information provided by high resolution reveal the fine relationships allow the direct visualization Improves contrast through metal staining	processed biological samples lower the resolution time consuming and expensive Poor contrast for biological systems Incompatible with 3D imaging Destructive technique	(167)
Mass spectrometry				
**ESI-MS**	Mitochondria targeting	Soft ionization Rapid analysis and automation Accuracy by multiple charging No matrix interferences	isolation requirment Destructive technique time consuming and expensive	(168)
**ICP-MS**	Mitochondria targeting	High sensitivity isotopic selectivity and a wide dynamic range Multi-element analysis Low sample volume needed High throughput	Detection limitation(ionization potential) isolation requirment time consuming and expensive Destructive technique Matrix interferences	(169)

Adapted from (94, 174, 182, 184, 192–195).

Inspired by natural dopamine, Li et al. synthesized polydopamine (PDA) nanoparticles, using triphenylphosphonium (TPP) for improved mitochondrial targeting efficiency, developing a mitochondria-targeted drug delivery system. By loading the anticancer drug doxorubicin (DOX) into PDA-polyethylene glycol (PEG) and TPP-functionalized PDA-PEG (PDA-PEG-TPP) nanoparticles, DOX can be effectively delivered to both the cell nucleus and mitochondria. Long-term repeated treatment of MDA-MB-231 cancer cells revealed that mitochondria-targeted PDA-PEG-TPP-DOX nanoparticles have a higher potential to overcome drug resistance than conventional delivery nanoparticles PDA-PEG-DOX (113). The higher pH of the mitochondrial matrix offers a specific trigger mechanism for drug release. For example, Zhang et al. constructed a novel mitochondria-targeted pH/ROS dual-responsive block copolymer TPP-PEG2k-b-(BS-AA)n (P1) and a non-targeted pH/ROS dual-responsive copolymer mPEG2k-b-(BS-AA)n (P2) for the stepwise release of chemotherapeutic drugs and simultaneous disruption of mitochondria and cell nuclei for combined anticancer chemotherapy. This system not only achieves controlled drug release in the specific mitochondrial environment but, through a unique response mechanism, directs the drug to mitochondrial DNA and nuclear DNA, enhancing the anticancer efficacy of chemotherapy drugs (114).

### 3.5 Transition metal complexes: mitochondrial targeting and photodynamic therapy potential

Transition metals like ruthenium (Ru) and iridium (Ir) complexes are found to possess innate mitochondrial targeting properties. These metal complexes can modulate their subcellular localization based on the type of ligands adjacent to their metal core and the counterions they carry, making them ideal photosensitizer candidates in photodynamic therapy due to their unique photophysical and photothermal properties (115), it also has great potential for building new diagnostic and treatment platforms (116). Guo et al. developed a mitochondria-targeted nanoplatform for near-infrared light-controlled nitric oxide release, coupled with photothermal therapy. This platform, consisting of nitroso-ruthenium functionalized nitrogen-doped graphene quantum dots and triphenylphosphonium components, demonstrated significant antitumor efficacy under 808 nm light irradiation both *in vitro* and *in vivo (* 117). However, their accumulation in mitochondria as lipophilic cationic-metal complexes may impair mitochondrial membrane potential, leading to cytotoxicity. Further discussions on mitochondria-targeted Ru and Ir-based nanomaterials are mentioned in related literature. Gu and colleagues developed a smart nanocomposite material composed of silver nanoparticles and polymer microspheres, designed for a dual attack on tumor cells: chemotherapeutic and enhanced oxidative stress response. Once inside the cell, the silver nanoparticles are released, activating internal chemotherapy drugs and inducing strong oxidative stress, leading to autophagic cell death of cancer cells with minimal impact on surrounding normal cells (118). Additionally, Deng and others explored a nanosystem composed of copper nanoparticles and porous carbon nanoparticles, which rapidly respond to intracellular environment changes upon entering tumor cells, releasing copper nanoparticles. This not only enhances the release efficiency of chemotherapeutic drugs but also significantly increases intracellular reactive oxygen species (ROS) levels, effectively inhibiting cancer cell growth while protecting normal cells (119). Zeng et al. further explored the application of metal-organic framework (MOF) materials in cancer therapy, finding that specifically designed MOFs can localize to mitochondria within cancer cells and persist for an extended period. By modulating mitochondrial membrane potential and promoting ROS generation, they effectively induce cancer cell death without harming normal cells. These results suggest that precisely designed nanomaterials can serve as a new generation of cancer therapy strategies, achieving targeted delivery and precise treatment (88).Thus, numerous studies focus on directly conjugating targeting ligands with therapeutics, aiming to enhance therapeutic efficacy (120).

## 4 Key factors in nanomedicine design and optimization for intracellular delivery

**Figure 5:**
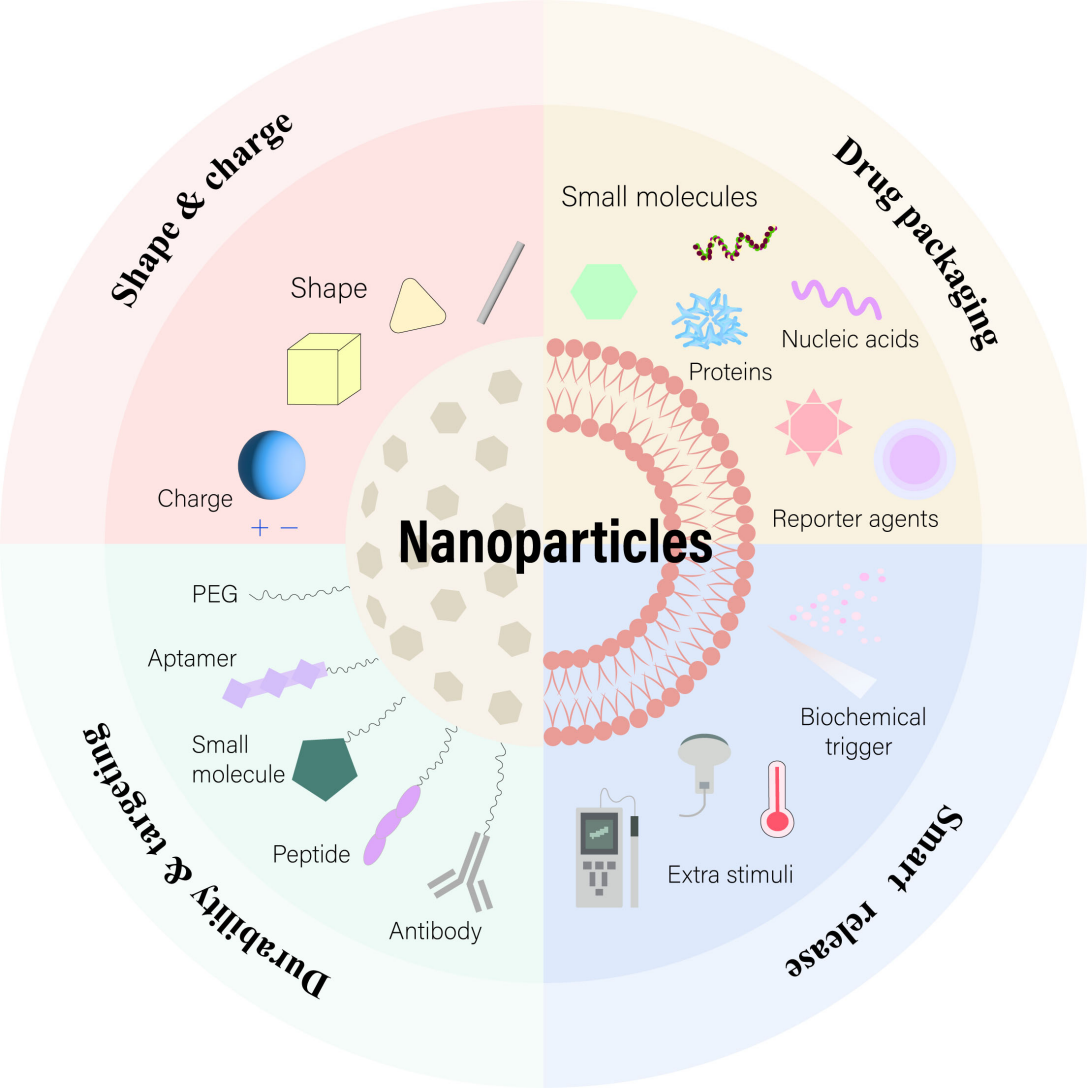
Nanoparticles with Enhanced Drug Delivery Properties. Surface modifications with PEG or targeting ligands improve nanoparticle stability and targeting. Adjusting morphology and surface charge optimizes their performance. At the target site, cargos are released through biochemical or external triggers. Adapted from (175).

The design of nanomedicines hinges on their physicochemical properties, such as size and shape, which affect their entry into cells via specific internalization pathways, while surface charge determines interactions with cells and toxicity (121, 122)(**Figure 5**). For instance, endothelial cells internalize particles of different sizes through specific mechanisms, with particle shape and charge affecting cellular uptake efficiency. Additionally, the physical stiffness of nanoparticles also impacts cell uptake preference, demonstrating inconsistent effects across different cell types (123). This underscores the necessity of considering these attributes in nanomedicine design to optimize intracellular delivery efficiency and reduce cytotoxicity.

### 4.1 Endosomal escape: a tool to aid the intracellular journey of nanomedicines

Employing chemical and physical strategies to facilitate endosomal escape and minimize cellular damage, the optimization of ionizable lipids that change charge states based on pH can enhance biocompatibility and circulation time. In acidic environments, these lipids become positively charged, promoting membrane fusion and endosomal rupture for effective nucleic acid delivery. The FDA and EMA-approved Onpattro^®^ and mRNA vaccines against SARS-CoV-2 demonstrate the medical application potential of this strategy (124). Although lipid nanoparticles have succeeded in delivering nucleic acids, endosomal escape remains a core challenge, with custom-designed lipid nanoparticles showing promise. To enhance cytoplasmic release efficiency of DNA, siRNA, and Cas9 mRNA in CRISPR/Cas gene editing, researchers developed a novel library of ionizable phospholipids. The library of ionizable phospholipids developed encompasses various structural types that can convert to a cationic state in acidic environments, interacting with endosomal membranes to facilitate hexagonal phase transitions and enhance cytoplasmic release (125–128). Specifically, these phospholipids consist of different head groups and fatty acid chain lengths, forming nanoparticles with varying solubility and charge properties (129). These nanoparticles are designed to interact with specific organelle membranes, allowing targeted delivery and efficient nucleic acid release (130–132). For example, Habrant and colleagues developed a series of ionizable carriers derived from the natural aminoglycoside antibiotic tobramycin, achieving diversity through structural optimization at the linker and hydrophobic domain levels. These carriers, forming complexes with mRNA, DNA, or siRNA, exhibited high transfection efficiency, particularly when combined with dual-stimuli (pH and near-infrared) controlled release, showing significantly higher drug release rates at acidic pH 5.5 compared to neutral pH 7.0, with further enhancement under acidic conditions (133). Another strategy involves using viral peptides with efficient endosomal escape capabilities, such as GALA peptide, which changes conformation in acidic environments to form transient pores, thus facilitating endosomal escape. Nakase et al.’s research shows the application of GALA peptide not limited to enhancing transfection efficiency of plasmid DNA mediated by cationic liposomes but also as a functional molecule in multifunctional enveloped nanodevices (MEND). Complexes of GALA with targeting molecules can effectively enter cells via endocytosis in the presence of cationic lipid complexes, successfully avoiding endosomal traps and potential metabolic degradation (134).

Most polymer-based nanomedicines achieve endosomal escape through the proton sponge effect, a mechanism involving the buffering action of polycationic polymers such as polyethyleneimine (PEI), peptides, chitosan, and other polymers, leading to increased osmotic pressure inside endosomes and causing them to rupture (135, 136). Specifically, BO-112, a ligand constructed from double-stranded RNA mimetics and PEI, is being used in clinical trials for melanoma, showcasing the application of the proton sponge effect in promoting cytoplasmic delivery. However, the understanding and application of the proton sponge mechanism are contentious, with studies suggesting other factors and mechanisms such as polymer conformation, shape, and degradation characteristics may play roles in endosomal escape. To address toxicity issues associated with traditional cationic nanomedicines, researchers have explored stimulus-responsive materials as alternatives to enhance the therapeutic effect of nanomedicine. A novel mRNA formulation encapsulated in a charge-reversal polymer that switches from negative to positive charge in acidic endosomal environments was developed. This formulation employs protonizable polyurethane derivatives (pHPUs) that promote endosomal escape through charge reversal at acidic pH, thereby enhancing mRNA delivery efficiency. The molecular design of pHPUs includes reversible protonizable groups that switch from a negative to a positive charge under acidic conditions, thereby enhancing mRNA delivery efficiency (137, 138). This design allows the polymer to respond to the acidic endosomal environment after cellular uptake, facilitating drug release into the cytoplasm and demonstrating safe and efficient endosomal escape functionality (139–141). Additionally, as alternatives to traditional endosomal escape mechanisms, strategies such as photochemical disruption and utilizing extracellular vesicles (EVs) as delivery vehicles have been proposed. Photochemical methods achieve effective drug or gene release by destroying endosomal membranes with reactive oxygen species (ROS) or thermal energy produced by photosensitizers. This strategy utilizes photosensitive materials like PEI-modified gold nanoparticles, which generate photothermal effects under near-infrared (NIR) irradiation, leading to endosomal membrane rupture and effective endosomal escape (142). Extracellular vesicles (EVs), as natural nanoparticles, offer an efficient delivery strategy by releasing their cargo through membrane fusion. Engineered EVs can encapsulate specific therapeutic molecules, which are taken up by target cells and release their load through membrane fusion, achieving targeted therapeutic effects. The advantage of this strategy lies in the natural origin and excellent biocompatibility of EVs, making them a promising therapeutic delivery system (143). These strategies have shown potential in promoting siRNA delivery in cancer models, although further optimization and preclinical studies are required. Combining different endosomal escape mechanisms in nanomedicine design may be key to achieving efficient cytoplasmic delivery.

### 4.2 Direct cell membrane penetration of customized nanoparticles

Direct cytoplasmic delivery of nanomedicines, bypassing the need for endosomal escape, can be achieved through membrane fusion or direct penetration. Membrane fusion is suitable for lipid-based nanomedicines, while direct penetration relies on the compactness, small size, cationic properties, or amphipathic surface patterning of the nanomedicine. Cell-penetrating peptides (CPPs) are extensively used to enhance the surface of nanomedicines for direct penetration, aiding in the delivery of nucleic acids, proteins, and drugs (144). CPPs, which include non-amphipathic cationic CPPs and primary and secondary amphipathic CPPs, are classified into cationic, amphipathic, and hydrophobic types (145). These CPPs facilitate membrane permeation by altering conformation or forming pores. Although the exact entry mechanisms of CPPs remain partially unknown, their efficiency is influenced by various factors such as concentration and cell type (146). Fine-tuning the physicochemical properties of nanomedicines, such as reducing size, surface functionalization with biodegradable CPPs, and designing amphipathic materials, contributes to direct cell membrane penetration and is an effective strategy for enhancing *in vivo* translation of nanomedicines (147, 148). To further enhance the efficiency of direct cytoplasmic delivery and bypass endocytosis, researchers have developed the following additional strategies: Firstly, the technique of “Membrane Perforation through Nanostructures” utilizes metallic nanoparticles to create temporary disruptions in the cell membrane, enabling direct delivery of proteins into the cytoplasm (149, 150). This includes methods such as using gold nanoparticles heated by a laser to induce membrane perforation (149). Secondly, “Membrane Translocation by Chemical Means” involves employing specially designed peptides and other chemical agents that interact with the cell membrane to create temporary openings or translocate directly through it. This approach includes the use of cell-penetrating peptides (CPPs) that can deliver cargo by penetrating the cell membrane without requiring endocytosis (151–154). Additionally, “Membrane Fusion Techniques” mimic viral entry mechanisms, where delivery vehicles fuse with the cell membrane, releasing their cargo directly into the cell (155, 156). This might involve the use of liposomes or engineered viral envelopes that can fuse with cellular membranes (157). Lastly, “Thermal and Mechanical Methods” apply physical forces or heat to disrupt the cell membrane temporarily, allowing proteins to enter directly. One example is sonoporation, where ultrasound-generated microbubbles are used to mechanically disrupt the cell membrane (158–160). These approaches are particularly advantageous for delivering molecules directly to the cytoplasm, bypassing the endosomal pathway which often leads to the degradation of therapeutic proteins (161, 162). During *in vivo* administration, nanocarriers must overcome numerous physiological barriers to reach the mitochondria, successfully release the drug, and exert the desired therapeutic effect. The overall charge of nanocarriers is crucial for their interaction with the biological environment. Positively charged nanocarriers tend to interact nonspecifically with blood components, leading to rapid clearance from the bloodstream (163). However, their positive charge is necessary for escaping from endolysosomes into the cytoplasm through the proton sponge effect (164). In contrast, negatively charged nanocarriers experience less serum protein adsorption but struggle to escape lysosomes. Employing a “stealth effect,” such as coating positively charged nanocarriers with hydrophilic materials like PEG, can prevent their adsorption by serum proteins and reduce nonspecific clearance in the body, thus prolonging circulation time and achieving passive targeting of tumor regions through the enhanced permeability and retention (EPR) effect. This section focuses on developing customized nanomedicines for precise subcellular targeting following cytoplasmic release, with a particular emphasis on mitochondrial delivery.

#### 4.2.1 Layered targeting for precision delivery

Layered targeting employs a multi-stage strategy, integrating various targeting elements and release mechanisms to ensure precise delivery to subcellular targets. Compared to charge-reversal materials, it enhances specificity and efficacy while minimizing off-target effects. Researchers developed a smart nanoparticle based on hyaluronic acid (HA) for tumor reductive response and CD44 receptor-mediated targeting therapy. These nanoparticles, formed by linking HA with poly(lactic-co-glycolic acid) (PLGA) through disulfide bonds, create a diblock copolymer (HAssLG). Doxorubicin (DOX)-loaded HAssLG nanoparticles prepared through dialysis exhibit a spherical appearance under 300 nm. Fluorescent measurements showed DOX release from nanoparticles in a dose-dependent manner upon adding glutathione (GSH), increasing with GSH addition, indicating their reductive responsiveness. The nanoparticles’ uptake by CD44-positive MDA-MB231 cells, confirmed through increased fluorescence upon treatment, was significantly reduced by pre-treatment with free HA to block CD44 receptors (165).

Moreover, a hyaluronic acid (HA)-modified micelle system was developed to encapsulate paclitaxel (PTX) and the P-glycoprotein inhibitor ritonavir (RTV). HA, a natural ligand for the CD44 receptor overexpressed in breast cancer cells, is conjugated to poly(lactic-co-glycolic acid) (PLGA) via a disulfide bond (HA-ss-PLGA), which is cleaved in the glutathione-rich environment of breast cancer cells. RTV inhibits P-gp and CYP3A4-mediated PTX metabolism, helping reverse multidrug resistance (MDR) and sensitize cells to PTX. *In vitro* studies showed greater uptake of micelles and PTX in breast cancer cell lines (MCF-7 and MDA-MB-231) compared to non-tumorigenic MCF-12A cells. Efficacy assays indicated reduced mitochondrial membrane potential and reactive oxygen species, promoting apoptosis in cancer cells (166).

Additionally, researchers have developed reversible crosslinked HA nanoparticles for targeted DOX delivery to CD44+ breast cancer cells using HA-Lys-LA conjugates (Lys: L-lysine methyl ester, LA: lipoic acid). DOX-loaded crosslinked nanoparticles formed by autogenous crosslinking exhibited inhibited DOX release under physiological conditions but rapid drug release in the presence of 10mM GSH. MTT tests showed that DOX-loaded crosslinked HA-Lys-LA10 nanoparticles had significant targeting and superior antitumor activity against DOX-resistant MCF-7 human breast cancer cells overexpressing CD44 receptors (MCF-7/ADR) (167). Additionally, a layered targeting strategy was employed using glucose transporter 1 (GLUT1) and matrix metallopeptidase 2 (MMP2) with PAMAM dendrimers for mitochondrial delivery of paclitaxel (PTX). The system, through MMP2-sensitive PEG layer detachment, GLUT1-mediated internalization, mitochondrial localization, and glutathione (GSH)-triggered PTX release, effectively reversed multidrug resistance (MDR) and enhanced the effect on MCF-7/MDR cells while reducing systemic toxicity, demonstrating potential in overcoming MDR in cancer treatment (168). Experimental findings indicated that the mitochondrial targeting conjugate, mediated by GLUT1 and triggered by MMP2, effectively overcame multidrug resistance in paclitaxel-resistant cancer cells, resulting in enhanced tumor suppression and reduced weight loss. Beyond cancer treatment, layered targeting strategies also extend to neurodegenerative diseases. For example, Sharma’s group proposed a mitochondrial targeting of hydroxy PAMAM dendritic drug constructs (TPP-D-NAC) by utilizing N-acetyl cysteine (NAC), where triphenyl-phosphonium (TPP) is used for mitochondrial targeting and NAC is used for targeting mitochondria delivered to injured glial cells. It was demonstrated that the dendrimer-NAC conjugate (D-NAC) significantly improved the attenuation of oxidative stress by TPP-D-NAC compared to free NAC (169).

#### 4.2.2 Enhancing specificity and efficacy of mitochondrial targeting

Delocalized lipophilic cations (DLCs), widely used as mitochondrial targeting molecules in nanomedicine, have facilitated the development of numerous preclinical nanodrugs but come with limitations. Their efficacy is compromised by post-treatment mitochondrial depolarization and inconsistent accumulation due to tumor cell heterogeneity. The concentration-dependent toxicity of DLCs and their nonspecific targeting across mitochondrial regions limit their broader application. To enhance specificity and effectiveness, research suggests combining mitochondrial targeting with specific disease markers. Sharma and colleagues developed a novel nanotherapeutic strategy by conjugating TSPO (translocator protein) targeting ligands to PAMAM (polyamidoamine) dendrimers through click chemistry, specifically for treating glioblastoma. With TSPO significantly upregulated in pathological conditions like glioblastoma, this strategy demonstrated specific targeting of tumor-associated macrophages and an enhanced anti-tumor immune response, representing an effective combination of mitochondrial and cellular specificity targeting, offering a new strategy for glioblastoma treatment (170). Due to the hearing impairment caused by aminoglycoside drugs leading to damage or loss of inner ear mechanosensory hair cells, and the significant role of mitochondrial cell death pathways in cellular dysfunction, a study developed a novel mitochondrial-targeting drug delivery system (DDS) to enhance the protective effect of gentamicin. This research successfully fabricated SS-31 peptide-conjugated geranylgeranylacetone (GGA)-loaded poly(lactic-co-glycolic acid) (PLGA) nanoparticles using the emulsion-solvent evaporation method. The results demonstrated that SS-31 conjugated nanoparticles exhibited mitochondrial specificity in hair cells accumulation; further experimental data suggested that the mitochondrial-targeting PLGA-based DDS has potential applications in protecting hair cells from ototoxic substances (171).

#### 4.2.3 Polymer carriers

Utilizing polymer carriers with mitochondrial targeting potential, such as amphiphilic polymers linked by disulfide bonds, shows advantages in cost-effectiveness and safety compared to other strategies. For example, Zhou and colleagues employed a charge reversal strategy to prepare lipid-polymer hybrid nanoparticles (LPNP) with triphenylphosphonium (TPP), using a polyethylene glycol (PEG) layer to conceal the positive charge of LPNPs for enhanced accumulation in tumor tissue. These LPNPs exhibited a nearly neutral ζ potential of +7.4 mV at pH 2.4 but regained a positive charge (ζ potential of +17.2 mV) upon encountering reduced glutathione (GSH) in cancer cells, causing the PEG layer to shed and allowing precise mitochondrial targeting. This design ensured the controlled release of paclitaxel (PTX) within mitochondria rather than the cytoplasm, enhancing anticancer efficacy (172). Similarly, the Momekova team developed a drug nanocarrier loaded with curcumin by combining two types of triblock copolymers—poly(ethylene glycol)-poly(ϵ-caprolactone)-poly(ethylene glycol) (PEG-PCL-PEG) and TPP-modified poly(2-(dimethylamino)ethyl methacrylate) (PDMAEMA)—to construct an initially positively charged outer layer, with the external PEG layer concealing the positive charge in the biological environment. Once internalized into cells, the PEG chains in lysosomal conditions degrade, exposing the positively charged core and facilitating lysosomal escape of the nanocarrier through the “proton sponge effect.” TPP-driven mitochondrial targeting release enhanced the cytotoxicity of curcumin (173).

In another example, a polymer developed by combining specific mitochondrial targeting ligands and dichloroacetate can activate mitochondrial oxidative stress to promote cell death in osteosarcoma models. Although the specific mechanism of mitochondrial affinity is not clear, adjusting the polymer properties can enhance cellular uptake and mitochondrial targeting. A study demonstrated that fluorinated amphiphilic molecules produced by the fluorination reaction of phosphatidylethanolamine (PE)-PEG and fluorocarbon chains (Fn) enhance the polymer’s cellular uptake and mitochondrial localization, showing potential for cancer treatment. Notably, PEG2k-F7 displayed exceptional cellular internalization abilities, with its potential-independent mitochondrial co-localization increasing drug efficacy. *In vitro* and in mouse models, PEG2k-F7 nanomicelles carrying anticancer drugs significantly reduced tumor size, confirming the application value of this mitochondrial targeting strategy in therapy (174). Zhang and colleagues developed a polymer nanomedicine design combining mitochondrial targeting, using long cyanine monomers (a family member of rhodamine DLCs) and dimethylacrylamide (DMA) copolymer, further functionalized with tumor-targeting cRGD peptide (targeting ανβ3 integrins) to prepare cRGD-P(DMA-co-CSMA) nanoparticles. These nanoparticles exhibited natural mitochondrial targeting through cyanine base-dependent accumulation without directly modified mitochondrial targeting ligands. In a melanoma model, dual-targeted nanoparticles loaded with the immunomodulator R848 precisely triggered mitochondrial damage and tumor antigen release through a mild photothermal effect, synergizing with PD-L1 immunotherapy to effectively inhibit primary and metastatic tumors, showcasing the potential of polymer nanomedicine in cancer therapy (175). This study highlights the potential of novel nanomedicine strategies combining mitochondrial targeting polymers and photothermal effects in enhancing cancer treatment efficacy, especially when used in conjunction with immunomodulators and PD-L1 inhibitors. By utilizing anionic polypeptide poly(γ-glutamic acid) (γ-PGA) and designed amphiphilic cationic β-sheet peptides, negatively charged peptide-peptide nanoparticles were successfully developed, effectively encapsulating the anticancer drug Lonidamine (LND) and demonstrating mitochondrial targeting capability. These peptide-based nanoparticles (LND-mPoP-NPs) increased LND loading by reducing peptide coating concentration, thus lowering nonspecific toxicity while retaining mitochondrial targeting ability. Further studies showed that this new formulation of nanoparticles (h-LND-mPoP-NPs), while maintaining a negative ζ potential, could effectively deliver drugs near the mitochondria. In tumor treatment applications, this optimized nanomedicine demonstrated stronger efficacy and tumor growth inhibition compared to the free drug (176). Additionally, another study designed and synthesized a new type of mitochondria-targeted multifunctional nanoparticle (MNPs) based on chitosan derivatives, achieving targeted release of anticancer drugs. These smart chitosan nanoparticles feature multiple functions such as stealth, hepatocyte targeting, multi-stage pH response, lysosomal escape, and mitochondrial localization. Guided by the tumor physiological environment, through stepwise shedding of functional groups, these nanoparticles effectively promote intracellular drug delivery and mitochondrial localization, enhancing antitumor effects while reducing the toxicity of anticancer drugs (16).

## 5 Current challenges and future directions in mitochondrial-targeted nanotherapeutics

### 5.1 Overcoming low drug loading efficiency

#### 5.1.1 Self-assembly of amphiphilic small molecules

To enhance drug encapsulation efficiency, some studies have adopted a “self-assembly” strategy involving the coupling of hydrophilic TPP with hydrophobic drugs to form amphiphilic TPP-drug derivatives. These derivatives self-assemble into nanoparticles at high concentrations, such as the TPP-PEG-biotin self-assembled nanoparticles successfully synthesized by Baskaran Purushothaman and colleagues, which enhanced stability, circulation time, and tumor tissue accumulation. These nanoparticles respond to cleavable PEG in the tumor microenvironment, allowing for disassembly without affecting cellular uptake and processing. *In vitro* and *in vivo* studies demonstrated that TPP-PEG-biotin nanoparticles, due to high mitochondrial accumulation, showed therapeutic effects surpassing that of free drugs, offering a straightforward method for the mitochondrial-targeted delivery of hydrophobic anticancer drugs (177). Furthermore, Khatun and others developed a bioreducible methoxy polyethylene glycol (mPEG)-triphenylphosphonium (TPP) conjugate-based bioreactive nanocarrier system, mPEG-(ss-TPP)2, as a vehicle for mitochondrial drug delivery. This amphiphilic mPEG-(ss-TPP)2 self-assembles in aqueous media to form core-shell structured nanoparticles (NPs) with good colloidal stability, efficiently encapsulating the hydrophobic anticancer drug doxorubicin (DOX). Studies on the anticancer efficacy and mitochondrial targeting capability of DOX-loaded mPEG-(ss-TPP)2 NPs indicated that bioreducible DOX-loaded mPEG-(ss-TPP)2 NPs could rapidly release the drug, enhance mitochondrial uptake, and achieve better therapeutic effects compared to non-bioreducible NPs (178). This showcases the potential of TPP-drug derivatives in improving drug encapsulation efficiency and achieving mitochondrial-targeted delivery, while also offering new avenues for therapeutic effects beyond traditional drug delivery methods.

#### 5.1.2 Self-assembly of small molecule-polymer conjugates

By coupling amphiphilic compounds like TPP-Dox with polymers, Khatun and colleagues demonstrated a strategy to avoid trapping these compounds within nanocarriers. This not only facilitated their self-assembly into mitochondria-targeted nanoparticles but also enhanced the uptake efficiency by tumor cells. They employed a bioreducible disulfide bond-based linking method to prepare polymers with mitochondrial targeting capability, forming a novel multifunctional bio-activatable mitochondria-targeted nanocarrier. These nanocarriers self-assembled in aqueous media, forming core-shell structured nanoparticles with good colloidal stability, effectively encapsulating the hydrophobic anticancer drug doxorubicin (DOX). The DOX-loaded nanoparticles exhibited rapid drug release and enhanced mitochondrial uptake capabilities, leading to better therapeutic effects than non-bioreducible nanoparticles (178). Additionally, Zhou and colleagues reported an improved strategy for enhancing the anticancer efficacy of paclitaxel through redox-triggered mitochondrial targeting. They designed a lipid-polymer hybrid nanoparticle (LPNP) containing TPP, with a PEG4000 layer on the surface to ensure high tumor accumulation. The nanoparticles regained surface charge under the reducing conditions inside cancer cells, achieving rapid and precise mitochondrial localization. This straightforward mitochondrial targeting nano-platform displayed high anticancer activity, offering a valuable strategy for the development of nanocarriers for other drugs (94).

### 5.2 Overcoming premature drug release

Wang and colleagues implemented an innovative strategy to address the issues of premature drug release and nonspecific distribution by synthesizing drugs *in situ* within mitochondria, thereby enhancing therapeutic efficiency and reducing toxicity. They utilized the copper-catalyzed azide-alkyne cycloaddition (CuAAC) reaction, initially creating a zirconium-based metal-organic framework (MOF) to stabilize copper nanoparticles (Cu NPs) as catalysts and introducing the mitochondrial targeting TPP moiety through amide coupling. Based on this, they conducted *in situ* synthesis using a resveratrol (Rsv) precursor, known to effectively induce apoptosis. This *in situ* synthesis method not only verified Rsv’s mitochondrial targeting effect within cells but also demonstrated significant antitumor activity with minimized side effects in a tumor-bearing mouse model (179). Additionally, another study explored a strategy of loading DNAzymes onto metal-organic frameworks (MOFs) as a new pathway for synchronizing *in situ* cancer drug synthesis with DNAzyme-based gene therapy. This bimetallic MOF was capable of releasing copper and zinc ions inside cancer cells to catalyze the CuAAC reaction for chemotherapeutic drug synthesis and activate the cleavage activity of DNAzymes, thus achieving precise cancer therapy (180).

## 6 Summary

**Figure 6:**
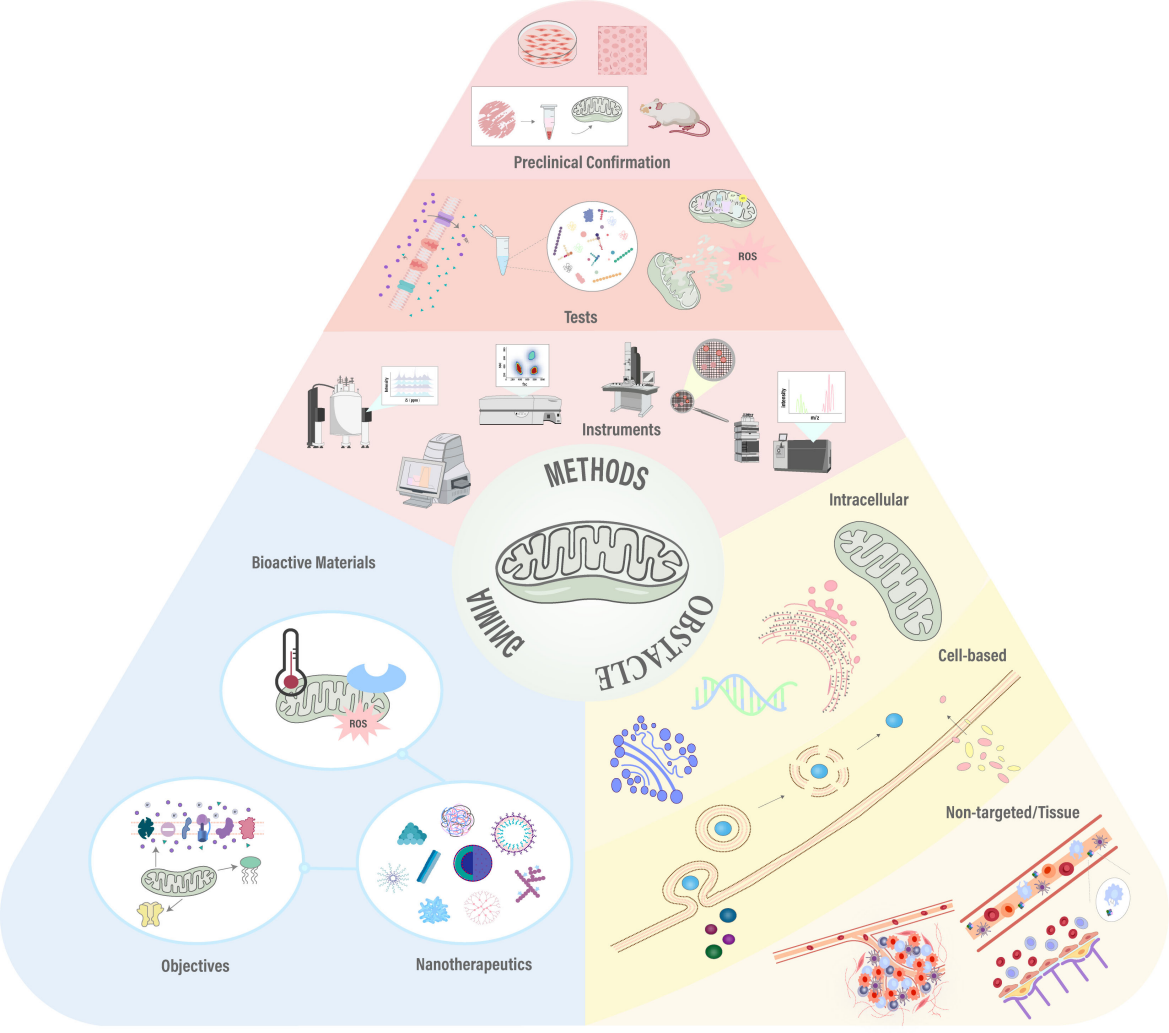
Graphical abstract: Progress in mitochondrial-targeted nanomedicine showcases overcoming biological barriers, strategic design, and subcellular validation techniques. Adapted from (154).

This review delves into the significant advancements in mitochondrial-targeted nanotherapeutics, an increasingly vital area for treating ailments associated with mitochondrial dysfunction, including cancer and neurodegenerative diseases. It discusses the latest developments in nanotechnology for drug delivery, focusing on the engineering of nanocarriers such as liposomes, polymer nanoparticles, and inorganic nanoparticles, all specifically designed for effective mitochondrial targeting. The review stresses the critical need to optimize the physicochemical properties of nanomedicines and to overcome biological barriers, thereby enhancing both delivery mechanisms and therapeutic outcomes (**Figure 6**). It also underscores the importance of developing non-toxic targeting ligands and employing innovative strategies that ensure site-specific drug release within mitochondrial compartments. Furthermore, the review advocates for the use of advanced physicochemical techniques to accurately assess mitochondrial dynamics and drug delivery efficacy. The incorporation of cutting-edge scientific methods such as metabolomics, proteomics, and machine learning is emphasized as essential for unraveling the complex mechanisms of action of mitochondrial-targeted therapies and for pushing the boundaries of their clinical applications. This comprehensive approach aims to pave the way for more precise and effective mitochondrial-targeted treatments in the future.
